# Meiotic cohesins mediate initial loading of HORMAD1 to the chromosomes and coordinate SC formation during meiotic prophase

**DOI:** 10.1371/journal.pgen.1009048

**Published:** 2020-09-15

**Authors:** Yasuhiro Fujiwara, Yuki Horisawa-Takada, Erina Inoue, Naoki Tani, Hiroki Shibuya, Sayoko Fujimura, Ryo Kariyazono, Toyonori Sakata, Kunihiro Ohta, Kimi Araki, Yuki Okada, Kei-ichiro Ishiguro

**Affiliations:** 1 Laboratory of Pathology and Development, Institute for Quantitative Biosciences, The University of Tokyo, Bunkyo-ku, Tokyo, Japan; 2 Department of Chromosome Biology, Institute of Molecular Embryology and Genetics, Kumamoto University, Chuo-ku, Kumamoto, Japan; 3 Liaison Laboratory Research Promotion Center, IMEG, Kumamoto University, Kumamoto, Japan; 4 Department of Chemistry and Molecular Biology, University of Gothenburg, Gothenburg, Sweden; 5 Department of Life Sciences, Graduate School of Arts and Sciences, The University of Tokyo, Meguro-ku, Tokyo, Japan; 6 Laboratory of Genome Structure and Function, the Institute for Quantitative Biosciences, University of Tokyo, Bunkyo, Tokyo, Japan; 7 Institute of Resource Development and Analysis & Center for Metabolic Regulation of Healthy Aging, Kumamoto University, Kumamoto, Japan; The University of North Carolina at Chapel Hill, UNITED STATES

## Abstract

During meiotic prophase, sister chromatids are organized into axial element (AE), which underlies the structural framework for the meiotic events such as meiotic recombination and homolog synapsis. HORMA domain-containing proteins (HORMADs) localize along AE and play critical roles in the regulation of those meiotic events. Organization of AE is attributed to two groups of proteins: meiotic cohesins REC8 and RAD21L; and AE components SYCP2 and SYCP3. It has been elusive how these chromosome structural proteins contribute to the chromatin loading of HORMADs prior to AE formation. Here we newly generated *Sycp2* null mice and showed that initial chromatin loading of HORMAD1 was mediated by meiotic cohesins prior to AE formation. HORMAD1 interacted not only with the AE components SYCP2 and SYCP3 but also with meiotic cohesins. Notably, HORMAD1 interacted with meiotic cohesins even in *Sycp2*-KO, and localized along cohesin axial cores independently of the AE components SYCP2 and SYCP3. *Hormad1/Rad21L*-double knockout (dKO) showed more severe defects in the formation of synaptonemal complex (SC) compared to *Hormad1-*KO or *Rad21L-*KO. Intriguingly, *Hormad1/Rec8*-dKO but not *Hormad1/Rad21L*-dKO showed precocious separation of sister chromatid axis. These findings suggest that meiotic cohesins REC8 and RAD21L mediate chromatin loading and the mode of action of HORMAD1 for synapsis during early meiotic prophase.

## Author summary

During meiotic prophase, homologous chromosomes undergo pairing/synapsis. HORMA domain-containing proteins (HORMAD1, HORMAD2) localize along unsynapsed chromosome axis and monitor homolog synapsis. However, molecular mechanisms how mouse HORMA domain-containing proteins that do not contain a definitive DNA-binding domain localize to the axis chromosome remains elusive. In particular, because chromosome axis is generated by meiotic cohesins (RAD21L, REC8) and axial element (AE) components (SYCP2, SYCP3), it has been elusive how those chromosome structural proteins contribute to the loading of HORMAD proteins onto the chromosomes. This was due to the unavailability of *Sycp2* KO mouse that should allow deep analysis of chromosome dynamics in the complete absence of AE. To solve this issue, in the present study, we newly generated *Sycp2* null mice, in which AE structure was completely abolished. This allowed us to assess meiotic cohesins and AE components separately, in terms of their roles on chromatin loading of HORMAD proteins. The most notable among our findings is that the localization of HORMAD1 on the chromatin is initially mediated through meiotic cohesins prior to axis formation. Intriguingly, our genetic study suggests that HORMAD1 in collaboration with meiotic cohesins plays distinct roles in sister chromatid cohesion, homolog synapsis and meiotic recombination during the early meiotic prophase.

## Introduction

Meiosis is a special mode of cell division that produces haploid gametes in sexually reproducing organisms. During meiotic prophase, homologous chromosomes (homologs) undergo pairing/synapsis, and meiotic recombination, yielding chiasmata whereby two homologs are physically connected [1–4].

Following pre-meiotic DNA replication at preleptotene, chromosomes are organized into proteinaceous structures, termed axial element (AE) or chromosome axis, which is assembled by the main components SYCP2 [5,6] and SYCP3 [7]. AE provides a scaffold for recruiting meiotic recombination machineries to promote double-strand break (DSB) introduction and repair [8,9] and for assembly of the synaptonemal complex (SC) [3,10,11].

Meiotic cohesin complex plays crucial roles not only in sister chromatid cohesion but also in axis formation, which underlies a structural basis for AE formation [12,13]. During meiotic prophase I, mitotic SCC1/RAD21-type cohesin is replaced with a meiosis-specific REC8-type [14,15] and RAD21L-type cohesins [16–18]. Chromatin loading of REC8 and RAD21L precede the loading of the main AE components SYCP2 and SYCP3 during early meiotic prophase, so that “cohesin axial core” is pre-formed and subsequently act as a framework to organize the AE [19]. REC8 and RAD21L show mutually exclusive localization along the axis, forming distinct cohesin-enriched domains [16,17]. Genetic studies demonstrated that REC8 and RAD21L play different roles in sister chromatid cohesion, DSB formation, meiotic recombination, and homolog pairing/synapsis [20–25]. Thus, it is assumed that these meiosis-specific chromosome events are exerted through specific actions of REC8 and RAD21L to the axis binding factors during early meiotic prophase. However, the interplay between distinct cohesin-enriched domains and axis components remained largely elusive.

In mice, meiotic recombination is initiated by the introduction of DSBs that are generated by SPO11 [26,27] and TOPO6BL [28,29]. HORMA domain-containing proteins **(**HORMAD1, HORMAD2**)** localize to unsynapsed axes and functions for the surveillance of asynapsis and the activation of ATR in unsynapsed regions [30–35]. HORMAD1 and its associated protein IHO1 plays an essential role in DSB formation by recruiting SPO11-accessory factors, REC114 and MEI4, to the axes [36,37]. It was hypothesized that HORMAD1/IHO1 [38] plays a role in tethering DSB hotspots marked by H3K4me3 and H3K36me3 to the axis through the interaction with methyltransferase PRMD9 [39–44] for the formation of DSB by SPO11/TOPO6BL complex. In yeast, Hop1 [45], a homolog of HORMAD1, localizes to the axis through Red1 and mediates DSB formation by recruiting Mei4/Rec114/Mer2 complex to the axis [46–48], suggesting the evolutionally conserved roles for HORMAD1/Hop1 in the initiation of meiotic DSBs.

The molecular mechanisms how mouse HORMAD1 and HORMAD2 that do not contain a definitive DNA-binding domain localize to the unsynapsed axis remain elusive. Although the HORMA domain of HORMAD2 directly interacts with SYCP2 *in vitro* through its N-terminus [49], it remained unknown whether HORMAD1 does. Previously, it was shown that HORMAD1 localization on the chromosome was retained in hypomorphic *Sycp2* mutant [6,31]. However, because truncated SYCP2 protein was still expressed in that hypomorphic *Sycp2* mutant [6,31], it remained elusive whether the HORMAD1 localization depended on SYCP2. Also in *Sycp3-*KO, where both SYCP3 and SYCP2 were absent from chromosomes [19], HORMAD1 still remained along the unsynapsed chromosomes [35]. Thus, it is yet to be addressed whether HORMAD1 localization depends on other axis-proteins besides the AE components SYCP2 and SYCP3. Further, it was not explored how HORMAD1 is loaded onto the chromosomes prior to axis development. Here we asked three questions (1) how HORMAD1 initially localizes onto the chromatin prior to axis development at early meiotic prophase, (2) whether HORMAD1 localization depends on other axis-proteins besides AE components (SYCP2 and SYCP3), and (3) whether meiotic cohesins have any role in HORMAD1 localization independently of AE components (SYCP2 and SYCP3). To address these issues, we newly generated *Sycp2* null mice, in which SYCP2 and SYCP3 were depleted from the chromosomes. This allowed us to assess meiotic cohesins and AE components separately, in terms of their roles on chromatin loading of HORMAD1 and HORMAD2. The most notable among our findings was that the localization of HORMAD1 on the chromatin was initially mediated through meiotic cohesins prior to axis formation, whereas SYCP2 stabilized the interaction between HORMAD1 and meiotic cohesins. We demonstrate that HORMAD1 interacts with both meiotic cohesins, RAD21L and REC8, *in vivo* and localizes along the “cohesin axial core” in the absence of AE components. Our comprehensive genetic analyses indicate that meiotic cohesins mediate the mode of action of HORMAD1 for synapsis. The present study highlights previously unforeseen modes of HORMAD1 function exerted through meiotic cohesins.

## Results

### HORMAD1 interacts with chromosome axis proteins and cohesins

**Fig 1:**
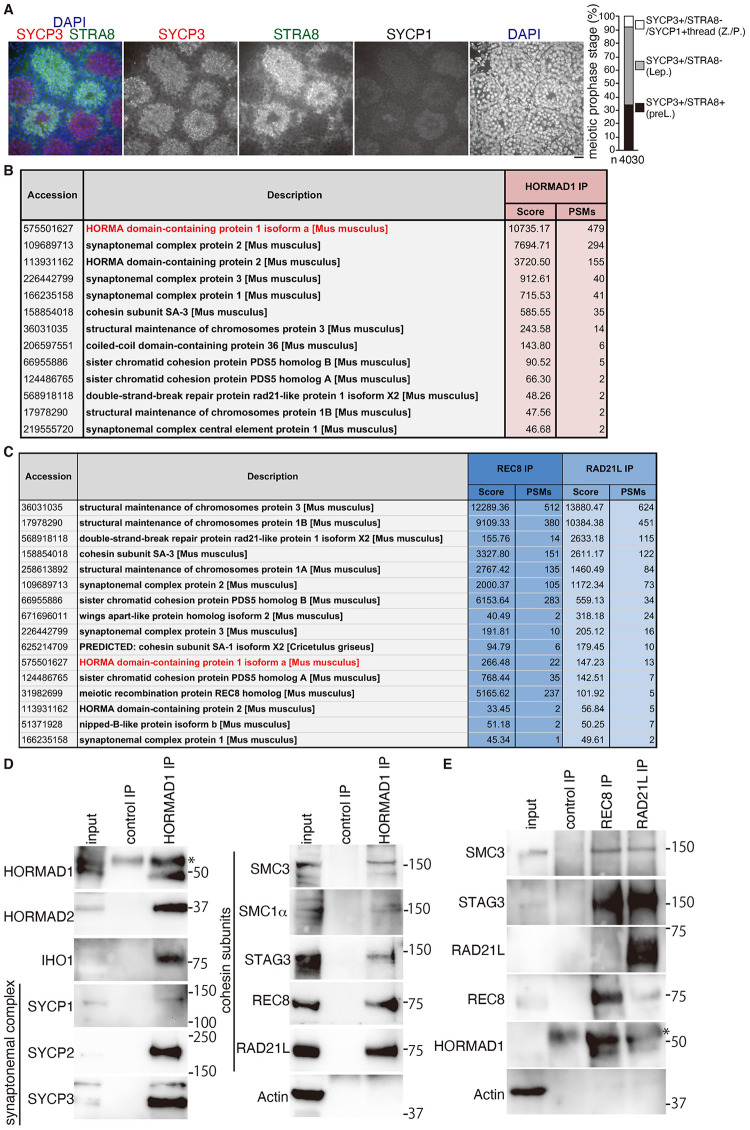
HORMAD1 interacts with cohesin and chromosome axis proteins. **(A)** WT neonatal male mice were subjected to consecutive injection of a RA synthesis inhibitor WIN18,446, followed by injection of RA. Seminiferous tubule section at 8 days post treatment were stained for SYCP3, STRA8, SYCP1(Cy5) and DAPI. Scale bar: 25 μm. Proportion of the meiotic prophase stages of spermatocytes is shown on the right. n: number of SYCP3 positive cells examined. preL: SYCP3+/STRA8+ preleptotene, Lep: SYCP3+/STRA8- leptotene. As weak staining of SYCP1 over spermatocyte nuclei was detected even in preleptotene and leptotene, spermatocytes with thread-like pattern of SYCP1 staining was defined as Z./P.: zygotene or pachytene. **(B)** The immunoprecipitates by anti-HORMAD1 antibody from the chromatin-bound fraction of the testis were subjected to liquid chromatography tandem-mass spectrometry (LC-MS/MS) analyses. The proteins identified by the LC-MS/MS analyses are listed with the number of peptide hits and Mascot scores. **(C)** The immunoprecipitates by anti-REC8 and anti-RAD21L antibodies from the chromatin-bound fraction of the testis were subjected to LC-MS/MS analyses as in (B). **(D)** WB analysis of HORMAD1 immunoprecipitates with indicated antibodies. HORMAD1 immunoprecipitates were run on 4–12% NuPAGE Bis-Tris in MOPS-SDS buffer. * indicates a band that cross-reacted to IgG heavy chain. **(E)** WB analysis of REC8 and RAD21L immunoprecipitates with indicated antibodies. REC8 and RAD21L immunoprecipitates were run on 4–12% NuPAGE Bis-Tris in MOPS-SDS buffer (for immunoblots of REC8, HORMAD1 and Actin) and 7% NuPAGE Tris-Acetate in Tris-Acetate-SDS buffer (for immunoblots of SMC3, STAG3 and RAD21L). * indicates a band that cross-reacted to IgG heavy chain.

HORMAD1 localizes along the unsynapsed chromosome axes during meiotic prophase. However, it remained elusive how HORMAD1 that does not contain a definitive DNA-binding domain is initially loaded onto the chromatin during early meiotic prophase. In order to elucidate the initial mode of HORMAD1 loading onto the chromatin during early meiotic prophase, we investigated HORMAD1-interacting proteins by immunoprecipitation (IP) of HORMAD1 from nuclear extracts of mouse testes. For this purpose, preleptotene/leptotene spermatocytes, whose chromosome axes labelled with SYCP3 were yet to be fully developed, were enriched by holding the development using WIN 18,446, an inhibitor of retinoic acid (RA) synthesis. Then the mice were injected with RA and the spermatocytes were collected 8 days after the injection [50] (Fig 1A). Chromatin extracts were prepared from testes, which were estimated to contain spermatocytes in preleptotene (~34%), leptotene (~56%) and SYCP1-thread+ zygotene/pachytene (~7.5%) (Fig 1A). Mass spectrometry (MS) analysis of the HORMAD1 immunoprecipitates identified chromosome axis proteins, in addition to the known interactors of HORMAD1 such as HORMAD2 [51] and IHO1[36] (Fig 1B and 1C and S1 Data). SYCP2 and SYCP3, the main components of AE, were substantially identified in the HORMAD1 immunoprecipitates. This indicated HORMAD1 directly or indirectly interacts with SYCP2 and SYCP3 *in vivo*, suggesting that the AE components SYCP2 and SYCP3 are among the primary axial proteins that binds to HORMAD1. It should be mentioned that SYCP1, a component of the transverse filament of SC, was also detected in HORMAD1 immunoprecipitates (Fig 1B and 1C), despite the enrichment of preleptotene/leptotene spermatocytes by WIN18,466. This is presumably due to extracts derived from the residual spermatocyte population that had reached zygotene when the testes were collected 8 days after RA injection (Fig 1A).

Notably, consistent with a previous study [35], cohesin subunits such as STAG3/SA3, SMC3, SMC1β, PDS5A, PDS5B and RAD21L were also detected in the MS analysis of HORMAD1 IP (Fig 1B), albeit with lower frequency. In mouse meiosis, there are two distinct types of meiosis-specific cohesin complexes, which possess either of the kleisin subunits REC8 or RAD21L. Therefore, we further analyzed the interaction of HORMAD1 with these meiosis-specific cohesin complexes. HORMAD1 IP followed by western-blot (WB) analysis further confirmed that REC8 and RAD21L as well as common cohesin subunits STAG3 and SMC3 were indeed immunoprecipitated with HORMAD1, suggesting that HORMAD1 interacts with both REC8- and RAD21L-cohesins (Fig 1D). Although SMC1β but not SMC1α was detected in the MS analysis of HORMAD1-IP (Fig 1B), by WB analysis SMC1α was detected in the HORMAD1-IP (Fig 1D). This suggested that small amount of SMC1α-containing cohesin, below the detection limit of MS analysis, interacted with HORMAD1. Reciprocal IP of REC8 and RAD21L followed by MS analysis further supported the above assumption that HORMAD1 interacted with both REC8- and RAD21L-cohesins (Fig 1C and 1E and S1 Data). Therefore, we hypothesized that HORMAD1 may have the ability to localize onto the chromatin through meiotic cohesins, either dependently or independently of SYCP2.

### HORMAD1 and HORMAD2 localize along the cohesin axial core in the absence of SYCP2

**Fig 2:**
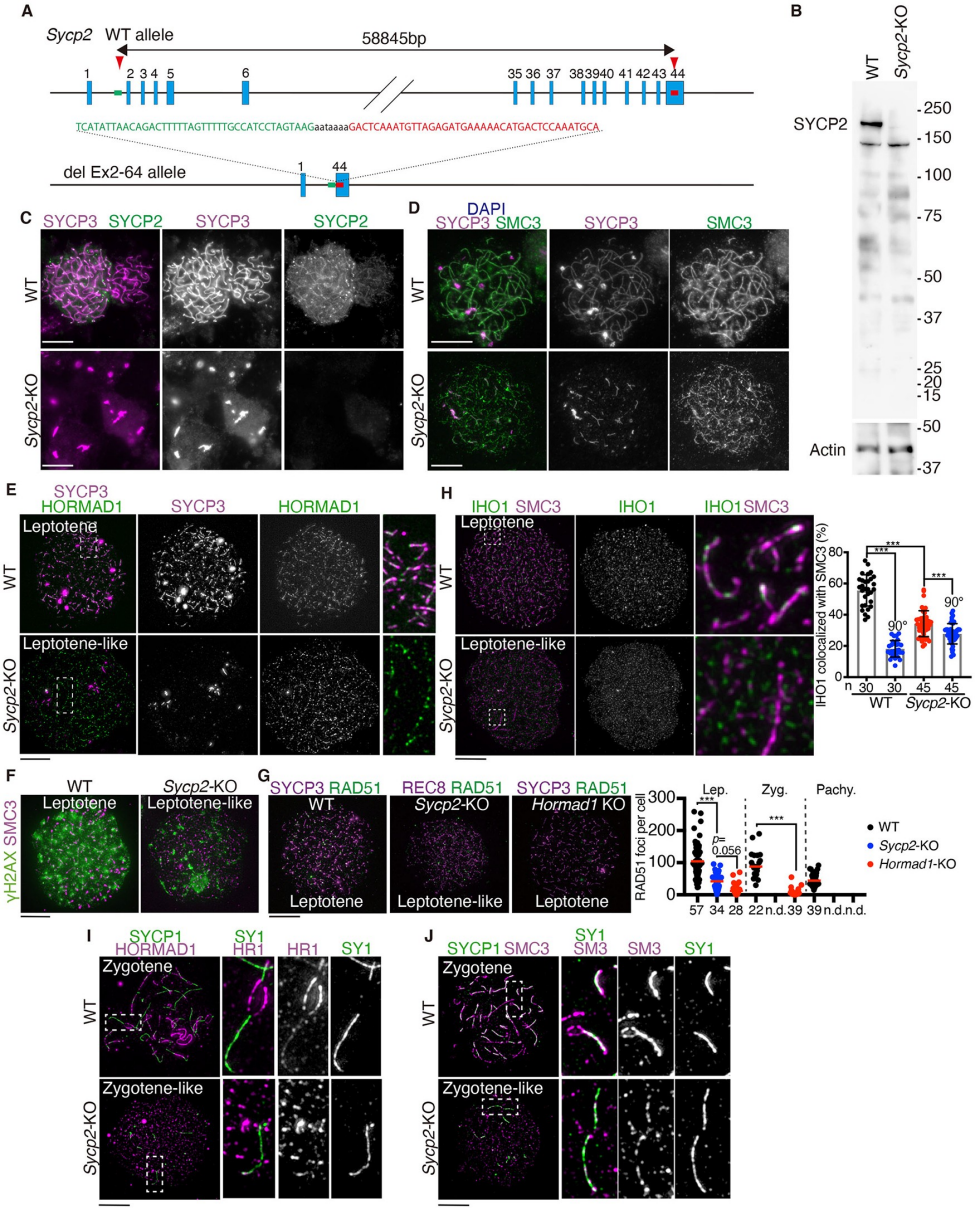
Generation of *Sycp2* complete null mouse. **(A)** The targeted Exon2-Exon44 deletion allele of *Sycp2* were generated by the introduction of CAS9, the synthetic gRNAs designed to target intron1 and Exon44 (arrowheads), and ssODN into C57BL/6 fertilized eggs. Two lines of KO mice were established. Line #17 of *Sycp2*-KO mice were used in most of the experiments, unless otherwise stated. **(B)** WB analysis of testis extracts prepared from WT and *Sycp2*-KO mice (P18). See also supplementary S1A Fig for immunoblots of other axis-associated proteins. **(C)** Chromosome spreads of WT and *Sycp2-*KO spermatocytes were stained for SYCP2 and SYCP3. Note that *Sycp2*-KO spermatocytes showed aggregation or short stretches of SYCP3. **(D)** Chromosome spreads of WT and *Sycp2-*KO spermatocytes were stained for SMC3 and SYCP3. Note that *Sycp2*-KO spermatocytes showed elongated stretches of SMC3, suggesting that cohesin axial core was generated in *Sycp2-*KO. **(E)** Chromosome spreads of WT and *Sycp2-*KO spermatocytes were stained for HORMAD1 and SYCP3. Enlarged images are shown on the most right. **(F)** Chromosome spreads of WT and *Sycp2-*KO spermatocytes were stained for SMC3 and γH2AX. **(G)** Chromosome spreads of WT, *Sycp2-*KO and *Hormad1*-KO spermatocytes were immunostained as indicated. Stage of *Sycp2-*KO spermatocytes was determined by REC8. The images of leptotene (-like) spermatocytes are shown (upper). The number of RAD51 dots per nuclei is shown in a scatter plot (right). The numbers of the cells analyzed are shown below the graph. Red bars indicate median. n.d.: not detected. Statistical significance was assessed by one-way ANOVA followed by Turkey’s multiple comparisons test with a single pooled variance. *: *P* < 0.05, **: *P* < 0.01, ***: *P* < 0.001. **(H)** Chromosome spreads of WT and *Sycp2-*KO spermatocytes were stained for SMC3 and IHO1. Enlarged images are shown on the right panels. Co-localization of IHO1 with SMC3 was quantified by counting IHO1 staining within 5 pixel distance from SMC3 threads (right). n: number of cells examined. To assess random overlaps, the co-localization was measured without (black and red plots) or with (blue plots) rotation of the IHO1 image by 90° clockwise relative to the SMC3 image. Statistical significance was assessed by one-way ANOVA followed by Turkey’s multiple comparisons test with a single pooled variance. **: *P* < 0.01, ***: *P* < 0.001. **(I)** Chromosome spreads of WT and *Sycp2-*KO spermatocytes were stained for SYCP1 and HORMAD1. Enlarged images are shown on the three right panels. Note that *Sycp2*-KO spermatocytes showed shortly elongated stretches of SYCP1, suggesting that homolog or non-homolog synapsis occurred in *Sycp2-*KO, but HORMAD1 was depleted from SYCP1-localized threads. **(J)** Chromosome spreads of WT and *Sycp2-*KO spermatocytes were stained for SYCP1 and SMC3. Enlarged images are shown on the three right panels. Note that SMC3 still localized along synapsed regions in *Sycp2*-KO spermatocytes. Scale bars: 10 μm. Note that because we cannot infer the exact stage from SYCP3 immunostaining pattern in *Sycp2-*KO, we refer to those spermatocytes, that exhibit stretched immunostaining pattern of cohesin (SMC3 or REC8) but does not show SYCP1, as leptotene-like stage spermatocytes.

Previous study showed that HORMAD1 localization remained on the chromosome in hypomorphic *Sycp2* mutant spermatocytes expressing truncated SYCP2 protein lacking the coiled coil domain [6,31]. HORMAD1 also remained on the chromosome in *Sycp3-*KO, in which SYCP2 together with SYCP3 was absent from the chromosome [35]. However, it still remained elusive whether HORMAD1 localization was independent of SYCP2 protein, because previous hypomorphic *Sycp2* mutant spermatocytes still expressed truncated SYCP2 protein that retained the putative HORMAD2 binding sites at the N-terminus. In order to precisely examine the impact on chromatin loading of HORMAD1 by loss of SYCP2, we generated *Sycp2* null mice. We deleted Exon2-Exon44 that includes the entire protein coding region of *Sycp2* loci in C57BL/6 fertilized eggs by the CRISPR/Cas9 system (Fig 2A). WB of *Sycp2-*KO testis showed that SYCP2 protein was completely absent (Fig 2B), indicating that the targeted *Sycp2* allele was null. In *Sycp2-*KO testis, the axis-associated proteins, HORMAD1, HORMAD2 and cohesins, were present comparably to WT, whereas SYCP3 protein was slightly reduced (S1A Fig). Histological analysis confirmed that the *Sycp2* null allele caused arrest of spermatogenesis, and as a consequence mature sperms were lost (S1A and S1B Fig) as expected. Although aggregations and short stretches labeled by SYCP3 were observed, continuous extended stretches of SYCP3 were absent in *Sycp2-*KO spermatocytes (Fig 2C). *Sycp2-*KO spermatocytes exhibited continuous stretches labeled by SMC3, a common subunit of cohesin (Fig 2D), suggesting that cohesin axial core was formed without SYCP2, which was similar to the previous observation in *Sycp3-*KO [19,35]. HORMAD1 exhibited dotty signals at early leptotene-like stage in *Sycp2*-KO (Fig 2E). DSBs labelled by γH2AX were detected at a lower level in *Sycp2*-KO compared to WT (Fig 2F). Accordingly, the number of RAD51 foci were markedly reduced in leptotene-like spermatocytes from *Sycp2*-KO compared to WT (Fig 2G), which was consistent with the recent study on zebra fish *Sycp2* mutant [5]. It is worth noting that despite the localization of HORMAD1 (Fig 2E), *Sycp2*-KO spermatocytes exhibited reduced number of RAD51 foci just like the *Hormad1*-KO spermatocytes (Fig 2G) [31–33,36,52]. Previously, it was shown that IHO1 appeared on chromatin at preleptotene, localized along the axis dependently on HORMAD1 and was required for efficient DSBs formation together with MEI4, REC114 and ANKRD31 [36,53,54]. IHO1 colocalized (~60%) along SMC3 in WT at leptotene, but less (~30%) in *Sycp2*-KO (Fig 2H), suggesting that axial localization of IHO1 was reduced in the absence of SYCP2. These observations suggested that DSB formation might be compromised in *Sycp2*-KO. SYCP1 were observed in *Sycp2-*KO spermatocytes (Fig 2I), suggesting that homologous or non-homologous synapsis progressed as has been shown in *Sycp3-*KO [19,35]. In *Sycp2-*KO, HORMAD1 localization was mutually exclusive to SYCP1 (Fig 2I) whereas SMC3 localization overlapped with SYCP1 (Fig 2J), suggesting that HORMAD1 dissociated from the synapsed regions of the cohesin axial core.

**Fig 3:**
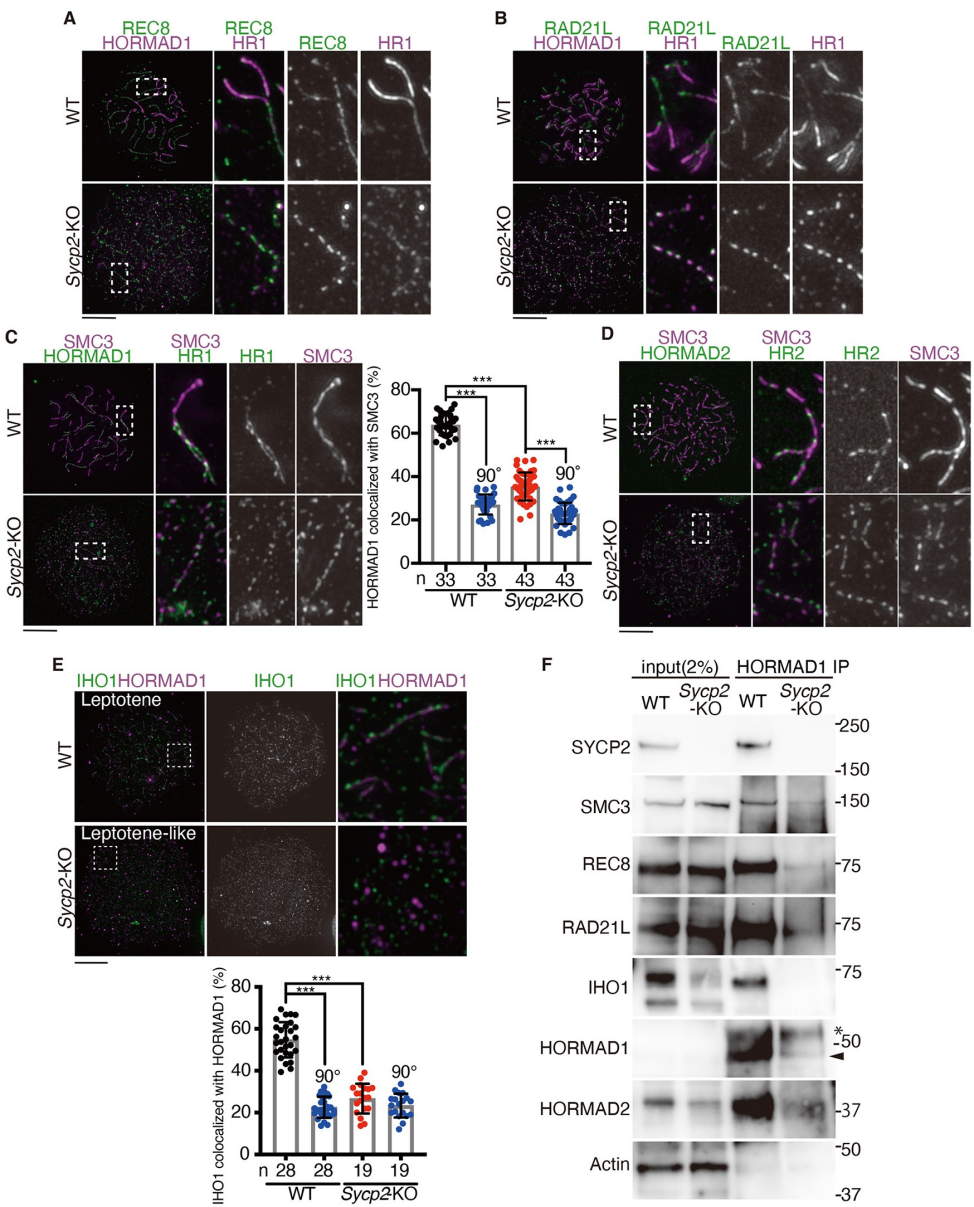
HORMAD1 localized along the cohesin axial cores in the absence of SYCP2. **(A)** Chromosome spreads of WT and *Sycp2-*KO spermatocytes were stained for HORMAD1 and REC8. Enlarged images are shown on the right. **(B)** Chromosome spreads of WT and *Sycp2-*KO spermatocytes were stained for HORMAD1 and RAD21L. Enlarged images are shown on the right. **(C)** Chromosome spreads of WT and *Sycp2-*KO spermatocytes were stained for SMC3 and HORMAD1 (upper). Enlarged images are shown on the right. Co-localization of HORMAD1 with SMC3 was quantified by counting HORMAD1 staining within 5 pixel distance from SMC3 threads. n: number of cells examined. To assess random overlaps, the co-localization was measured without (black and red plots) or with (blue plots) rotation of the HORMAD1 image by 90° clockwise relative to the SMC3 image. Statistical significance was assessed by one-way ANOVA followed by Turkey’s multiple comparisons test with a single pooled variance. ***: *P* < 0.001. **(D)** Chromosome spreads of WT and *Sycp2-*KO spermatocytes were stained for SMC3 and HORMAD2. **(E)** Chromosome spreads of WT and *Sycp2-*KO spermatocytes were stained for IHO1 and HORMAD1 (upper). Enlarged images are shown on the right. Co-localization of IHO1 with HORMAD1 was quantified as in (C). **(F)** HORMAD1-immunoprecipitates from WT and *Sycp2*-KO testis extracts were probed with indicated antibodies. HORMAD1-immunoprecipitates were run on 4–12% NuPAGE Bis-Tris in MOPS-SDS buffer. * indicates a non-specific band. Arrowhead indicates the band of HORMAD1. Scale bars: 10 μm.

Notably, despite the absence of SYCP2, these dotty signals of HORMAD1 emerged along the cohesin axial core labeled by either RAD21L or REC8 in leptotene-like *Sycp2-*KO spermatocytes (Fig 3A and 3B). Around 40% of HORMAD1 staining localized along the cohesin axial core labeled by SMC3 in leptotene-like *Sycp2-*KO spermatocytes, while WT leptotene spermatocytes showed more than 60% co-localization (Fig 3C), suggesting that axial localization of HORMAD1 was partly reduced in the absence of SYCP2. HORMAD2 also localized along the cohesin axial core labeled by SMC3 in leptotene-like *Sycp2-*KO spermatocytes (Fig 3D). Colocalization of IHO1 with HORMAD1 was significantly reduced in *Sycp2-*KO spermatocytes (Fig 3E), suggesting that IHO1-HORMAD1 interaction depends on SYCP2. Crucially, the cohesin subunits SMC3, REC8 and RAD21L were detected in HORMAD1-IP in *Sycp2-*KO extracts (Fig 3F), suggesting that HORMAD1 had the ability to interact with meiotic cohesins independently of SYCP2. We noticed that HORMAD2 was detected in HORMAD1-IP in *Sycp2-*KO extracts, suggesting that HORMAD1 interacted with HORMAD2 independently of SYCP2. IHO1 was reduced in *Sycp2-*KO chromatin fraction and was not detected in HORMAD1-IP from *Sycp2-*KO chromatin extracts, supporting the above assumption that DSB formation might be compromised in *Sycp2*-KO (Fig 2H). It should be mentioned that HORMAD1 was detected in HORMAD1 IP at a lower level in *Sycp2-*KO compared to WT (Fig 3F). Since the expression of HORMAD1 was comparable in *Sycp2-*KO and WT (S1A Fig), maybe HORMAD1 protein became less soluble in *Sycp2-*KO and did not bind to the HORMAD1-antibody used for this IP. When the IP-products were normalized with HORMAD1 detected in HORMAD-IP, the amount of meiotic cohesins and HORMAD2 bound to HORMAD1 were comparable in *Sycp2-*KO and WT. Altogether, these results suggested that HORMAD1 and HORMAD2 localized to unsynapsed cohesin axial core independently of SYCP2.

### HORMAD1 is in close proximity to meiotic cohesin before axis formation

**Fig 4:**
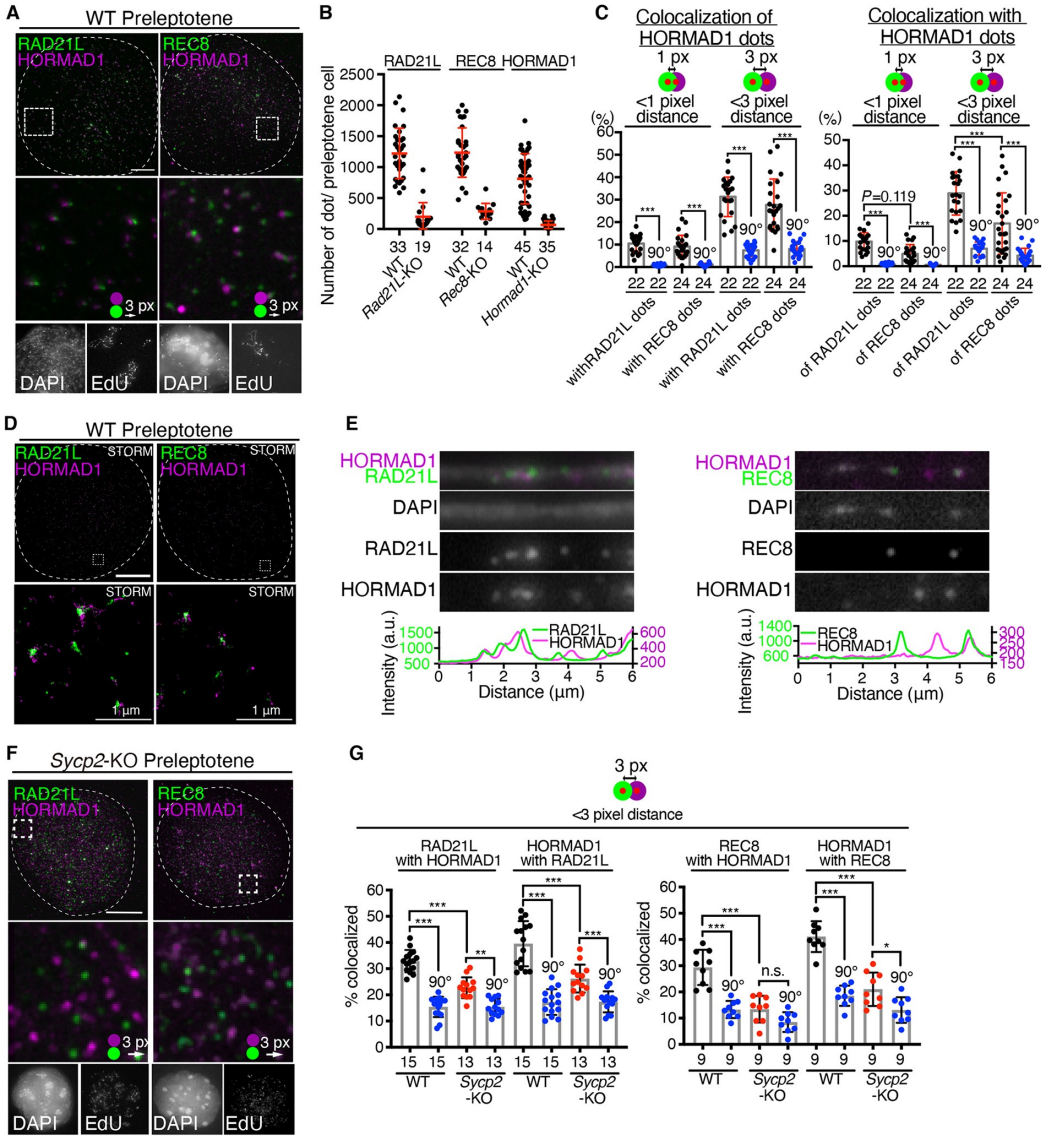
Partial colocalization of HORMAD1 with meiotic cohesins. **(A)** HORMAD1 was co-immunolabelled with RAD21L or REC8 on spread chromatin of WT preleptotene spermatocytes (upper). For visualization, green channels were shifted to the right by three pixels in the magnified views (middle). Preleptotene cells were identified by patchy EdU labelling pattern that represents late S phase (lower small panel) and by punctate cohesin staining. Note that slight difference in the localization pattern of RAD21L on the chromatin have been observed among previous studies [15,16,18,21]. This difference could be due to the sensitivity and/or cross reactivity with RAD21 of the antibodies used. **(B)** The number of the immunolabelled dotty signals of RAD21L, REC8 and HORMAD1 are shown in scatter plots. The numbers of the cells observed are shown below the graph. Red bars indicate mean and SD. **(C)** Quantification of HORMAD1 dots that overlapped with RAD21L or REC8 dots (left), and RAD21L or REC8 dots that overlapped with HORMAD1 dots (right) are shown in bar graphs with SD. To show these were not random overlaps, the co-localization was measured without (black plot) or with (blue plot) rotation of the HORMAD1 image by 90° clockwise relative to the cohesin images. Co-localization was measured by ComDet plugin for ImageJ (see Methods). Co-localization was assessed by two criteria, either < 1 pixel distance or < 3 pixel distance. Statistical significance was assessed by one-way ANOVA followed by Turkey’s multiple comparisons test with a single pooled variance. ***: *P* < 0.001. The numbers of the cells observed are shown below the graph. Red bars indicate SD. **(D)** Super-resolution imaging of spread chromatin using N-STORM microscopy showed that HORMAD1-enriched domains (magenta) and the cohesins RAD21L- and REC8-enriched domains (green) were localized side by side. Scale bars: 10 μm unless indicated. **(E)** Immunolabeling of HORMAD1, REC8 and RAD21L on stretched chromatin fibers of preleptotene/leptotene WT spermatocytes confirmed that HORMAD1, REC8 and RAD21L were loaded onto the chromatin. DNA fiber was labelled with DAPI. **(F)** HORMAD1 was co-immunolabelled with RAD21L or REC8 on spread chromatin of *Sycp2*-KO preleptotene spermatocytes (upper). For visualization, green channels were shifted to the right by three pixels in the magnified views (middle). Scale bars: 10 μm. **(G)** Quantification of RAD21L dots that overlapped with HORMAD1 dots (left), and HORMAD1 dots that overlapped with RAD21L dots (right) in WT and *Sycp2*-KO preleptotene spermatocytes are shown as in (C) (left graph). Quantification of REC8 dots that overlapped with HORMAD1 dots (left), and HORMAD1 dots that overlapped with REC8 dots (right) in WT and *Sycp2*-KO preleptotene spermatocytes are shown (right graph). Co-localization was assessed by < 3 pixel distance. Statistical significance was assessed by one-way ANOVA followed by Turkey’s multiple comparisons test with a single pooled variance. * *P* < 0.05. ** *P* < 0.01. ***: *P* < 0.001. The numbers of the cells analyzed are shown below the graph. n.s.: not specific. Back bars indicate mean and SD.

In meiotic prophase, REC8- and RAD21L-cohesins appear on the chromatin prior to AE formation and show mutually exclusive cytologically recognized domains [16]. Given that HORMAD1 interacts with both REC8- and RAD21L-cohesins and localizes along the cohesin-axis, the distances between HORMAD1 and RAD21L or REC8 on spread chromatin of preleptotene spermatocytes were assessed. Preleptotene spermatocytes were identified by dotty cohesin signals that represent RAD21L or REC8, and patchy EdU labelling pattern that represents late S phase [36] (Fig 4A). In WT spermatocytes, RAD21L, REC8 and HORMAD1 exhibited punctate signals at preleptotene and early leptotene, whose median number ranged around 500–1500 per nucleus (Fig 4B). Those dots were diminished in *Rad21L*-KO, *Rec8*-KO and *Hormad1*-KO mice, respectively (see also Fig 6), indicating that they represented *bona fide* localization of those proteins rather than immunostaining backgrounds. These observations imply that HORMAD1 and cohesins are initially enriched on a limited number of cytologically recognized domains rather than being localized evenly on the whole chromatin. Notably, ~ 10% of RAD21L dots and ~ 5% of REC8 dots were juxtaposed to those of HORMAD1 within 1 pixel proximity during preleptotene (Fig 4C), and this fraction increased to ~ 30% of RAD21L dots and ~ 15% of REC8 dots when they were counted for 3 pixel proximity of HORMAD1 (Fig 4C). Conversely, ~ 10% and ~ 9% of HORMAD1 dots were juxtaposed to RAD21L and REC8 dots within 1 pixel proximity, respectively, and ~ 31% and ~27% within 3 pixel proximity, respectively (Fig 4C). Further, the super-resolution single molecule localization analysis using STORM microscopy demonstrated that the HORMAD1 dots, if not all, were juxtaposed closely to RAD21L- and REC8-enriched domains (Fig 4D), consistent with aforementioned assumption. Indeed, linear scanning of immuno-localization signals along the extended fibers of the chromatin indicated that HORMAD1 associated with RAD21L and REC8 on the chromatin (Fig 4E). In preleptotene *Sycp2*-KO spermatocytes, juxtaposition of HORMAD1 to RAD21L dots was reduced compared to WT (Fig 4F and 4G). Also, juxtaposition of HORMAD1 to REC8 dots in preleptotene *Sycp2*-KO spermatocytes was reduced to a level close to random overlap (Fig 4F and 4G). These results suggested that association of HORMAD1 with cohesins might be stabilized by SYCP2.

**Fig 5:**
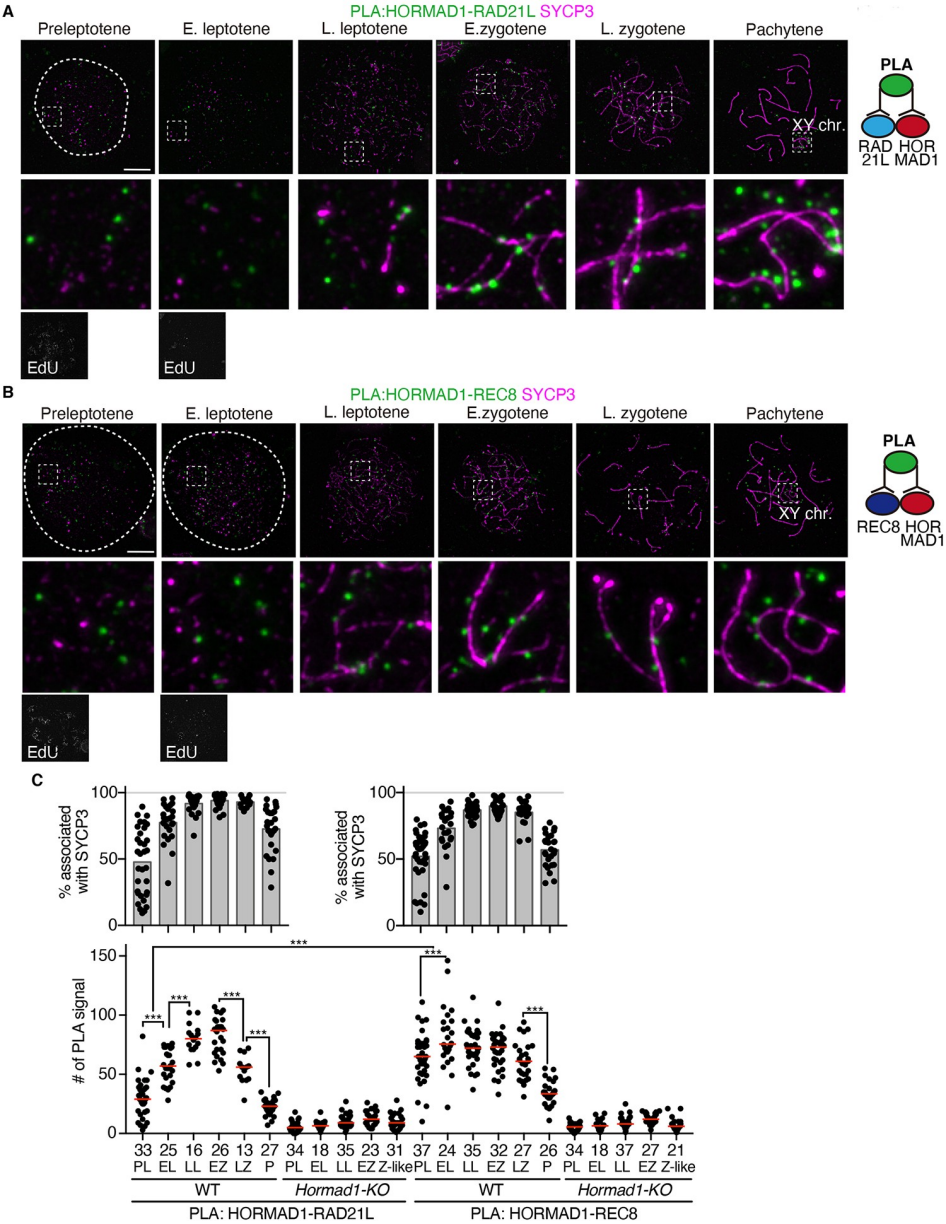
HORMAD1 is in close proximity to meiotic cohesins. **(A, B)** *In situ* protein-protein proximity between HORMAD1 and RAD21L **(A)** or REC8 **(B)** were analyzed by Proximity Ligation Assay (PLA) shown in green. SYCP3 (magenta) was immunolabelled to analyze the association of PLA signals with the axis. **(C)** Scatter plot shows the number of PLA signals per nucleus at different stages during meiotic prophase I (Lower). *Hormad1*-KO cells were used as a negative control to assess the specificity of the PLA signals. Quantification of PLA signals that were associated with SYCP3 is shown on the upper graph. Bar graphs indicate average % of PLA signals associated with SYCP3 threads (upper). N: the number of the cells observed. Statistical significance was assessed by one-way ANOVA followed by Turkey’s multiple comparisons test with a single pooled variance. ***: *P* < 0.001. Scale bars:10 μm.

To further examine whether immuno-colocalization of HORMAD1-RAD21L and HORMAD1-REC8 at preleptotene through leptotene represented spatial proximity of those proteins, protein interactions were assessed by *in situ* Proximity ligation assay (PLA) on spread chromatin. In WT spermatocytes, PLA foci for HORMAD1-RAD21L were detected throughout meiotic prophase I (Fig 5A). Those foci were largely diminished in *Hormad1*-KO background (Fig 5C), confirming the specificity of the PLA assay. The number of PLA foci for HORMAD1-RAD21L culminated before early zygotene and declined after zygotene stage (Fig 5C), probably due to the dissociation of HORMAD1 from the synapsed axis [30–33,35]. Crucially, PLA foci for HORMAD1-RAD21L were detected as early as in preleptotene, of which appreciable fraction (~50%) were apart from SYCP3-stained signals (Fig 5B and 5C). Thus, HORMAD1 and RAD21L were indeed in close proximity on the chromatin before axis formation. Similarly, PLA foci for HORMAD1-REC8 were detected throughout meiotic prophase I (Fig 5B and 5C). PLA foci for HORMAD1-REC8 were detected as early as in preleptotene, of which ~50% fraction were apart from SYCP3-stained signals. Thus, HORMAD1 and REC8 were indeed in close proximity on the chromatin before axis formation. These observations suggested that the primary loading of HORMAD1 on the chromatin was mediated by RAD21L- and REC8-cohesins before axis formation.

### Meiotic cohesin is required for chromatin loading of HORMAD1 during early meiotic prophase

**Fig 6:**
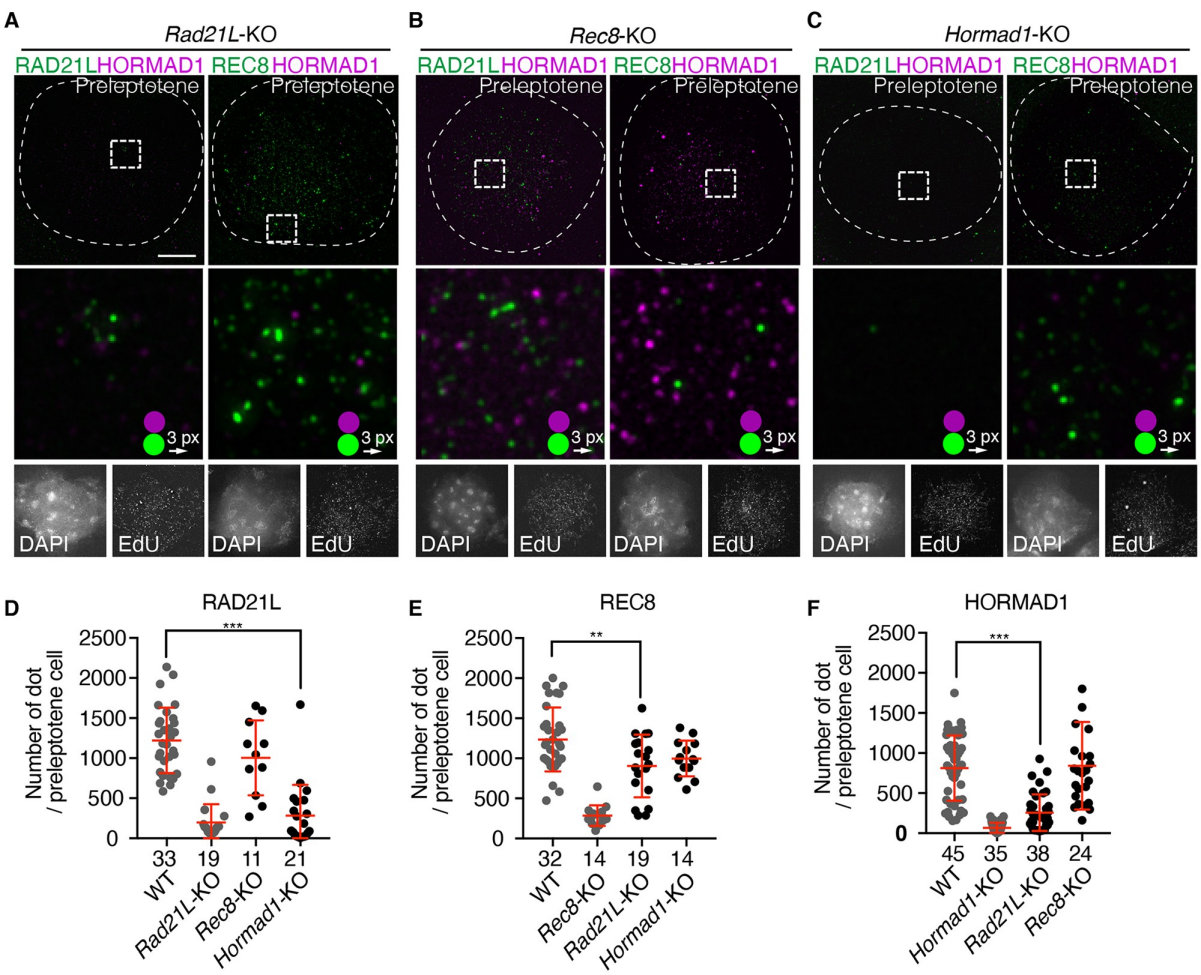
Meiotic cohesins are required for initial chromatin loading of HORMAD1 during early prophase. (**A—C**) Spread chromatin of preleptotene spermatocytes from *Rad21L*-KO **(A)**, *Rec8*-KO **(B)**, and *Hormad1*-KO **(C)** mice were immunolabelled for HORMAD1 (magenta), RAD21L (green) and REC8 (green). (**D-F**) Scatter plots show the number of immunolabelled HORMAD1 dots in a preleptotene cell. Red lines represent mean and SD. N: the number of the cells observed. Statistical significance was assessed by one-way ANOVA followed by Turkey’s multiple comparisons test with a single pooled variance. *: *p* < 0.05, ***: *P* < 0.001.

Lacking any one of the meiotic cohesin subunits, RAD21L, REC8, STAG3 or SMC1β partly affects AE formation and synapsis, and lacking both RAD21L and REC8 affects severely [13,21–23,25,55–58]. To examine whether RAD21L and REC8 are required for the localization of HORMAD1 onto the initial loading domains, HORMAD1 was immunostained on the spread chromatins of *Rad21L*-KO and *Rec8*-KO preleptotene spermatocytes. Remarkably, in *Rad21L*-KO mice the number of HORMAD1-enriched domains in preleptotene spermatocytes was significantly reduced (Fig 6A and 6F), while it was less affected in *Rec8*-KO mice (Fig 6B and 6F). These observations suggested that, at least in part, RAD21L may play a major role in the initial loading of HORMAD1 onto the chromatin in preleptotene. As the exact protein level of HORMAD1 in *Rec8-*KO and *Rad21L*-KO was not examined, it is also possible that HORMAD1 expression was reduced in these mutant testis. We noticed that RAD21L loading was affected by *Hormad1*-KO at this stage (Fig 6C and 6D) but not at later stage, suggesting that RAD21L loading was delayed in the absence of HORMAD1. These suggest that HORMAD1-RAD21L interaction may stabilize each other for chromatin loading.

It should be mentioned that HORMAD1 eventually localized along the AE at later stage of meiotic prophase in *Rad21L*-KO and *Rec8*-KO (S2 and S3 Figs). Because HORMAD1 localization along the cohesin axial core is reduced and become discontinuous in *Sycp2*-KO (Fig 2) and in *Sycp3*-KO [35,59], it is plausible that AE components stabilize the interaction of HORMAD1 with meiotic cohesins, and as a consequence the axis-localization of HORMAD1 is stabilized. Therefore, these results suggest that chromatin loading of HORMAD1 may be regulated initially via meiotic cohesins prior to axis development, which is subsequently stabilized by AE components.

### Meiotic cohesins mediate the mode of action of HORMAD1 for SC formation

HORMAD1 and HORMAD2 play roles in chromosome synapsis and DSB formation [30–36]. Given that HORMAD1 was enriched at close proximity to meiotic cohesins in early prophase (Figs 4 and 5), we assessed how HORMAD1 exerts chromosome synapsis in *Rad21L*-KO or *Rec8*-KO.

**Fig 7:**
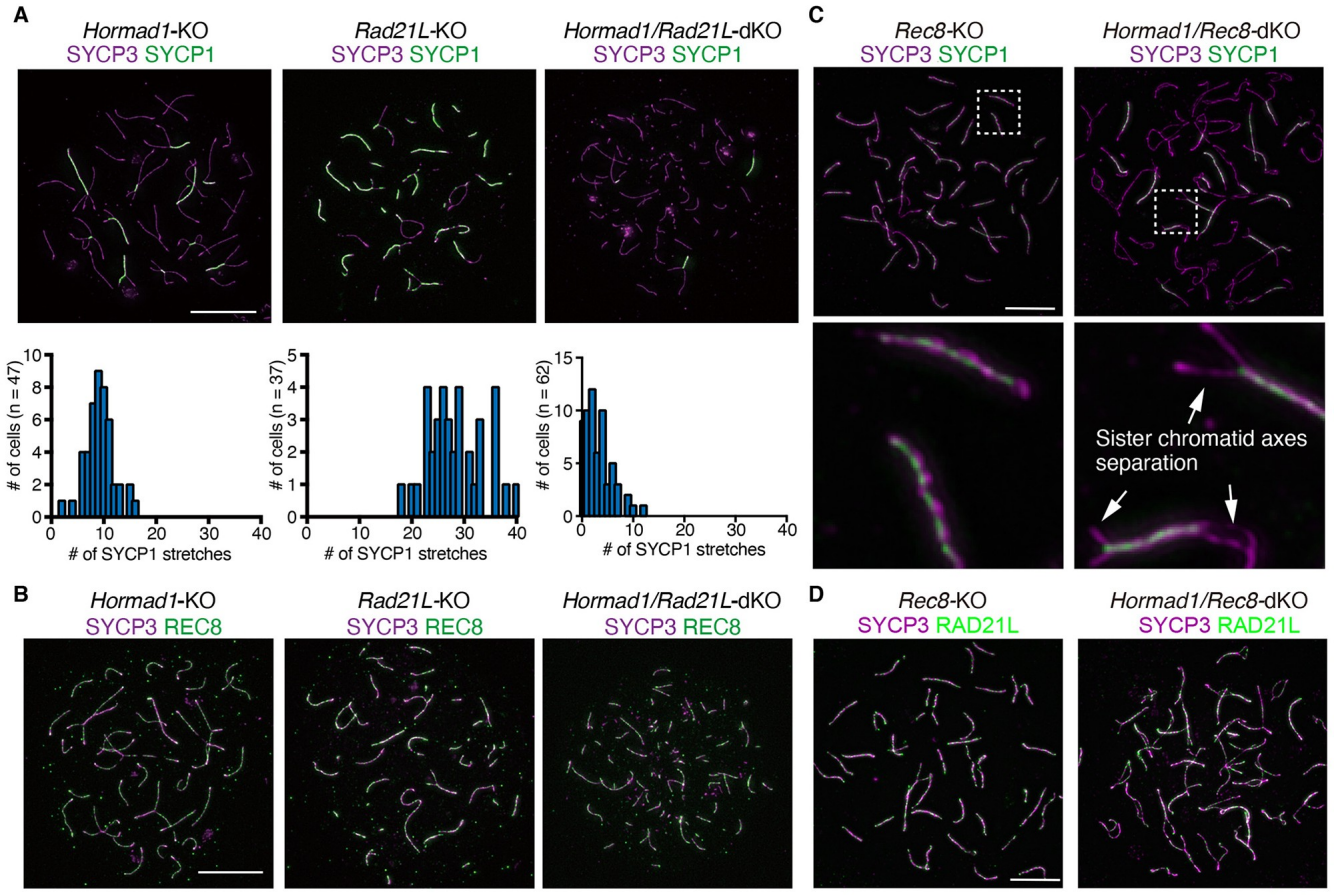
HORMAD1 promotes RAD21L-dependent inter-sister SC formation. **(A)** Spread chromatin of zygotene-like spermatocytes from *Hormad1*-KO, *Rad21L*-KO and *Hormad1/Rad21L*-dKO were immunolabelled for SYCP3 (magenta) and SYCP1 (green). The frequency distribution of SYCP1 stretches are shown on the bottom. n indicates the number of the spermatocytes analyzed. **(B)** Spread chromatin of zygotene-like spermatocytes from *Hormad1*-KO, *Rad21L*-KO and *Hormad1/Rad21L*-dKO were immunolabelled for SYCP3 (magenta) and REC8 (green). **(C)** Spread chromatin of zygotene-like spermatocytes from *Rec8*-KO and *Hormad1*/*Rec8*-dKO were immunolabelled for SYCP3 (magenta) and SYCP1 (green). Enlarged images are shown on the bottom. Arrows indicate sister chromatid axis separations. **(D)** Spread chromatin of zygotene-like spermatocytes from *Rec8*-KO and *Hormad1*/*Rec8*-dKO were immunolabelled for SYCP3 (magenta) and RAD21L (green). Scale Bars: 10 μm.

In *Rad21L/Hormad1*-dKO background, homologous or non-homologous chromosome synapsis were substantially impaired in zygotene-like stage compared to *Rad21L*-KO and *Hormad1*-KO (Fig 7A), suggesting that HORMAD1 synergistically acts for SC formation with RAD21L.

Previously it was demonstrated that sister chromatid axes were locally separated and tethered by inter-sister SC in REC8-free regions during the most advanced zygotene-like stage in *Rec8*-KO [21–23], *Stag3-*KO and *Smc1β*-KO spermatocytes [60]. It was proposed that the high density of randomly distributed REC8-cohesin promotes sister chromatid cohesion and prevents illegitimate SC formation [60], while RAD21L-cohesin plays a minor role in sister chromatid cohesion [12,21]. Intriguingly, in *Hormad1*/*Rec8*-dKO but not in *Rec8*-KO, sister chromatid axes were precociously separated in zygotene-like stage (Fig 7C) despite the localization of RAD21L along the axes (Fig 7D). This phenotype was in sharp contrast to that observed in *Hormad1*/*Rad21L*-dKO (Fig 7A), where REC8 localized along normally tethered sister chromatid axes (Fig 7B). Therefore, these observations suggested that inter-sister synapsis in the absence of REC8 depended on both HORMAD1 and RAD21L. It should be mentioned that precocious separation of sister chromatid axes was similarly observed in *Spo11/Rec8*-dKO that lacked DSBs [12,21]. Because HORMAD1 plays a role in DSB formation [30–37] and RAD21L was the sole cohesin in *Rec8*-KO spermatocytes (Fig 7D), maybe RAD21L-dependent inter-sister SC was established through HORMAD1-mediated DSBs.

## Discussion

### Meiotic cohesins mediate initial chromatin loading of HORMAD1 during early meiotic prophase

The present study showed that initial chromatin loading of HORMAD1 is mediated by meiotic cohesins before and during axis development. HORMAD1 was initially enriched on a limited number of cytologically recognized domains of RAD21L and REC8 during preleptotene (Fig 4). Initial chromatin loading of HORMAD1 was mediated at least in part by RAD21L (Fig 6A). In contrast, genetic analysis using *Rec8*-KO suggested that REC8 plays a minor role in this process at preleptotene (Fig 6B), despite some fraction of HORMAD1 was in close proximity to REC8 at preleptotene. Then one question is raised: why are DMC1 foci essentially not affected in *Rad21L*-KO [20,21] despite the decrease of HORMAD1 in the absence of RAD21L. Although the exact reason for this paradox is unknown, it is possible that whereas REC8 is dispensable for the initial loading of HORMAD1 onto the chromatin, REC8-cohesin may eventually compensate the loading of HORMAD1 in *Rad21L*-KO because HORMAD1 has eventually localized along the axis in *Rad21L*-KO (S2 and S3 Figs). We observed reduced initial loading and delayed localization of RAD21L to the axis in the absence of *Hormad1* (Fig 6D). This implies that HORMAD1 also directly or indirectly mediates RAD21L loading through an unknown mechanism. Although the exact reason is yet to be clarified, it is possible that RAD21L might be loaded in a DSB-dependent manner as has been shown in budding yeast SCC1-cohesin [61].

Although MS analysis suggested that SYCP2 substantially associates with HORMAD1 (Fig 1B), SYCP2 is dispensable for the initial interaction between HORMAD1 and meiotic cohesin, as shown by the establishment of the interaction in *Sycp2*-KO background (Fig 3). Since the co-localization of HORMAD1 and meiotic cohesins were detected at lower levels in *Sycp2*-KO compared to those in WT (Fig 3), it is possible that the AE component SYCP2 may play a role in stabilizing HORMAD1-cohesin interaction. The mode of chromosome localization of HORMAD1 and HORMAD2 in mouse is partly concordant with the model in yeast. In yeast meiotic prophase, Rec8 is the sole kleisin subunit of cohesin and the axis localization of Hop1, the yeast homolog of HORMAD1, requires Rec8 [46] and an axis component Red1/Rec10 [62,63]. In mouse, meiotic cohesins are required for initial chromosome localization of HORMAD1 and HORMAD2 independent of SYCP2 (Figs 2 and 3). In addition, mouse SYCP2 is required for robust production of DSBs in addition to the presence of HORMAD1 (Fig 2F), which resembles the yeast model where Red1/Rec10 is required to produce normal level of DSBs [63,64].

### HORMAD1 collaborates with RAD21L and REC8 for SC formation

It has been proposed that REC8 establishes sister chromatid cohesion in a DNA-replication-dependent manner, whereas RAD21L does probably in a DSB-dependent manner and less contributes to cohesion [12,21]. In *Rec8*-KO, where RAD21L is the sole cohesin, sister chromatid axes were tethered by inter-sister synapsis rather than canonical sister chromatid cohesion. Notably, we showed that sister chromatid axes were separated in *Hormad1*/*Rec8*-dKO (Fig 7D), which was similar to the phenomena observed in *Spo11*/*Rec8*-dKO [12,21]. In contrast, in *Hormad1*/*Rad21L*-dKO where REC8 is the sole cohesin, sister chromatid axes were intact (Fig 7B). Implication for these observations is that inter-sister SC formation observed in *Rec8*-KO depends on RAD21L and HORMAD1. Because HORMAD1 promotes DSB formation [36,52], RAD21L-dependent inter-sister SC formation observed in *Rec8*-KO may be established in the vicinity of HORMAD1/RAD21L-enriched domains.

It has been proposed that HORMAD1, collaborating with IHO1, promotes SPO11-mediated DSB by recruiting PRDM9/CXXC1-containing complex from the hotspot DNA loop to the axis [38]. Another study also suggested that REC8 preferentially associates with PRDM9 on the DNA loop rather than the axis during early prophase [65]. Thus, it is possible that HORMAD1/REC8-enriched domains may promote DSB hotspot formation by recruiting PRDM9 containing complex during early prophase. Given that inter-sister SC observed in *Hormad1*/*Rec8*-dKO **(**Fig 7) was similar to those in *Spo11*/*Rec8*-dKO [12,21], it is plausible that HORMAD1 promotes DSB-dependent inter-sister synapsis and recombination. This idea is partly consistent with a genetic study showing that HORMAD2 plays a role in inhibiting inter-sister recombination-mediated DSB repair [66]. Altogether, HORMAD1 may play two distinct roles in SC formation and DSB formation through the action of meiotic cohesins.

## Materials and methods

### Animal experiments

*Sycp2* (KO) mice were C57BL/6 background. *Hormad1-*KO mice [33], *Rad21L*-KO mice [21], *Rec8* (*mei8*) mutant mice [23] (although the *mei8* allele has a point mutation, here it is referred to as *Rec8*-KO mice), *Spo11*-KO mice (*Spo11*^*tm1Mjn*^) [26], were congenic with the C57BL/6 background, and were used for cytological experiments. Mice of the C57BL/6J strain were obtained from Japan SLC, Inc. (Shizuoka, Japan). Each knockout animal was compared with littermates or age-matched (12–25 dpp) non-littermates from the same colony. No statistical method was used to estimate sample size.

Animal experiments were approved by the Institutional Animal Care and Use Committee (approval #2715, #2809 at The university of Tokyo, approval F28-078, A30-001, A2020-006 at Kumamoto University). All methods and animal protocols were carried out in accordance with institutional animal guidelines and regulations.

### Generation of *Sycp2* knockout mice and genotyping

*Sycp2* knockout mouse was generated by introducing Cas9 protein (317–08441; NIPPON GENE, Toyama, Japan), tracrRNA (GE-002; FASMAC, Kanagawa, Japan), synthetic crRNA (FASMAC), and ssODN into C57BL/6N fertilized eggs using electroporation. For generating *Sycp2* Exon 2–44 deletion (Δ Ex2-44) allele, the synthetic crRNAs were designed to direct TTTGCCATCCTAGTAAGTGT (TGG) of the *Sycp2* intron 1 and TCTCTAACATTTGAGTCGGG (AGG) in the Exon44 of the *Sycp2*. ssODN: 5’-TGCATTTGGAGTCATGTTTTTCATCTCTAACATTTGAGTCtttattCTTACTAGGATGGCAAAAACTAAAAAGTCTGTTAATATGA -3’ was used as a homologous recombination template.

The electroporation solutions contained [10 μM of tracrRNA, 10 μM of synthetic crRNA, 0.1 μg/μl of Cas9 protein, ssODN (1 μg/μl)] for *Sycp2* knockout in Opti-MEM I Reduced Serum Medium (31985062; Thermo Fisher Scientific). Electroporation was carried out using the Super Electroporator NEPA 21 (NEPA GENE, Chiba, Japan) on Glass Microslides with round wire electrodes, 1.0 mm gap (45–0104; BTX, Holliston, MA). Four steps of square pulses were applied (1, three times of 3 mS poring pulses with 97 mS intervals at 30 V; 2, three times of 3 mS polarity-changed poring pulses with 97 mS intervals at 30 V; 3, five times of 50 mS transfer pulses with 50 mS intervals at 4 V with 40% decay of voltage per each pulse; 4, five times of 50 mS polarity-changed transfer pulses with 50 mS intervals at 4 V with 40% decay of voltage per each pulse).

The targeted *Sycp2* Ex2-44Δ allele in F0 mice were identified by PCR using the following primers; *Sycp2*-F1: 5’-GCAAAGATGCATCATCTAAGCACCTC-3’ and *Sycp2*-R1: 5’- CAAAGTATGAAGGTACTAACAATTAAAG-3’ for the knockout allele (459 bp). *Sycp2*-F1 and *Sycp2*-R2: 5’- ATACTTGGAGGTCTGGTCTCACTGGC-3’ for the Ex2 up stream of wild-type allele (582 bp). *Sycp2*-F2: 5’-GCAAAGATGCATCATCTAAGCACCTC3’and *Sycp2*-R1 for the Exon 44 of wild-type allele (354 bp). The PCR amplicons were verified by sequencing.

*Sycp2* knockout mouse lines generated in this study have been deposited to Center for Animal Resources and Development (CARD, ID 2881).

### Preparation of testis extracts and immunoprecipitation

To enrich preleptotene and leptotene spermatocytes, male mice were injected daily with WIN 18,446 (14018, Cayman Chemical, MI, USA) during 3–11 dpp, followed by RA injection at 12 dpp and testes collection after 8 days of the treatment [50,67]. Twenty or more testes (from 10 or more animals) per one IP were collected and pooled for IP-MS experiments. Although we did not check every testis that was collected for the preparation of testis extracts, enrichment of preleptotene/leptotene population was assessed by immunostaining in a given testis sample among the pooled testes. For immunoprecipitation from WT and *Sycp2*-KO mice, *Sycp2*-KO testes were pooled from 7 animals and corresponding control testes were collected from WT (10 dpp) mice. Testis chromatin-bound extracts were prepared as described previously [21]. Briefly, testicular cells were suspended in low salt extraction buffer (20 mM Tris-HCl [pH 7.5], 100 mM KCl, 0.4 mM EDTA, 0.1% TritonX100, 10% glycerol, 1 mM β-mercaptoethanol) supplemented with Complete Protease Inhibitor (Roche). After homogenization, the soluble chromatin-unbound fraction was separated after centrifugation at 100,000 × *g* for 30 min. The chromatin bound fraction was extracted from the insoluble pellet by high salt extraction buffer (20 mM HEPES-KOH [pH 7.0], 400 mM KCl, 5 mM MgCl_2_, 0.1% Tween20, 10% glycerol, 1 mM β-mercaptoethanol) supplemented with Complete Protease Inhibitor. The solubilized chromatin fraction was collected after centrifugation at 100,000 × *g* for 30 min at 4 °C.

2.5 μg of affinity-purified rabbit anti-HORMAD1 and control rabbit IgG antibodies **(**Table 1**)** were crosslinked to 50 μl of protein A-Dynabeads (10002D, Thermo Fisher Scientific, USA) by DMP (D8388, Sigma-Aldrich, USA). The antibody-crosslinked beads were added to the testis chromatin-bound extracts prepared from wild type testes. The beads were washed with high salt extraction buffer. The bead-bound proteins were eluted with 40 μl of elution buffer (100 mM Glycine-HCl [pH 2.5], 150 mM NaCl), and then neutralized with 4 μl of 1 M Tris-HCl [pH 8.0]. The immunoprecipitated proteins were run on 4–12% Bis-Tris NuPAGE (NP0322, Thermo Fisher Scientific) in MOPS-SDS buffer or 7% Tris-Acetate NuPAGE (EA030552, Thermo Fisher Scientific) in Tris-Acetate-SDS buffer for immunoblotting or LC-MS/MS analysis. Immunoblots were detected by VeritBlot for IP detection reagent (HRP) (ab131366, abcam, 1:3000 dilution). For repeated reprobing the immunoblots, immobilon-PVDF membranes (Merck), that were cut at the appropriate molecular weight, sequentially immunoblotted and antibodies were striped using Restore Western Blot Stripping Buffer (Thermo 21059). Immunoblot image was developed using ECL prime (RPN2232, GE healthcare) and captured by FUSION Solo (VILBER) or LAS4000mini (GE healthcare, USA).

**Table 1:** Antibodies used.

REAGENT or RESOURCE	SOURCE	IDENTIFIER	Dilution (IF)	Dilution (WB)
Antibodies				
rabbit anti-HORMAD1	ProteinTech	13917-1-AP	1:500	1:1000
rabbit anti-HORMAD2	[34]		1:1000	1:1000
mouse anti-HORMAD1	This study		1:500	
mouse anti-HORMAD2	This study		1:200	1:500
mouse anti-RAD21L	[16]		1:1000	
rabbit anti-RAD21L	[16]		1:500	1:1000
rabbit anti-RAD51	Santa Cruz	sc-8349	1:50	
mouse anti-REC8	[16]		1:1000	
rabbit anti-REC8	[16]		1:500	1:1000
rat anti-SYCP3	[71]		1:1000	
rabbit anti-SYCP1	Abcam	ab15090	1:200	1:1000
Mouse anti-SYCP1	[16]		1:200	
Mouse anti-SMC1α	[16]			1:1000
rabbit anti-SYCP3	Abcam	ab15093	1:500	1:1000
guinea pig anti-SYCP3	[67]		1:2000	
mouse anti-SA3	[16]		1:1000	1:500
rabbit anti-SMC3	Abcam	ab9263	1:1000	1:1000
mouse anti-SYCP2-C	This study		1:1000	
rabbit anti-SYCP2-C	This study		1:500	1:1000
mouse anti-IHO1	This study			1:500
mouse anti-gammaH2A.X(S139)	Abcam	ab26350	1:2000	
rabbit anti-gammaH2A.X(S139)	Abcam	ab11174	1:2000	
rabbit anti-ACTIN	CST	#4970		1:1000
VeriblotBlot for IP detection reagent (HRP)	Abcam	ab131366		1:3000
Donkey anti-mouse IgG CF 568	Biotium	20105	1:500 for STORM	
Donkey anti-mouse IgG-Alexa Fluour 488	Thermo Fisher Scientific	A21202	1:1000	
Donkey anti-rabbit IgG-Alexa Fluour 488	Thermo Fisher Scientific	A21206	1:1000	
Goat anti-mouse IgG-Alexa Fluour 568	Thermo Fisher Scientific	A11004	1:1000	
Goat anti-rabbit IgG-Alexa Fluour 568	Thermo Fisher Scientific	A11011	1:1000	
Goat anti-rat IgG-Alexa Fluour 568	Thermo Fisher Scientific	A11077	1:1000	
Donkey anti-mouse IgG-Alexa Fluour 647	Thermo Fisher Scientific	A31571	1:1000	
Goat anti-rabbit IgG-Alexa Fluour 647	Thermo Fisher Scientific	A21244	1:1000 (1:500 for STORM)	
Goat anti-rat IgG-Alexa Fluour 647	Thermo Fisher Scientific	A21247	1:1000	

### Identification of HORMAD1 associated factors by mass spectrometry

The immunoprecipitated proteins were run on 4–12% NuPAGE (Thermo Fisher Scientific) by 1 cm from the well and stained with SimplyBlue (LC6065, Thermo Fisher Scientific) for the in-gel digestion. The gel containing proteins was excised, cut into approximately 1mm sized pieces. Proteins in the gel pieces were reduced with DTT (20291, Thermo Fisher Scientific), alkylated with iodoacetamide (90034, Thermo Fisher Scientific), and digested with trypsin and lysyl endopeptidase (Promega, USA) in a buffer containing 40 mM ammonium bicarbonate, pH 8.0, overnight at 37 °C. The resultant peptides were analyzed on an Advance UHPLC system (AMR/Michrom Bioscience) coupled to a Q Exactive mass spectrometer (Thermo Fisher Scientific) processing the raw mass spectrum using Xcalibur (Thermo Fisher Scientific). The raw LC-MS/MS data was analyzed against the NCBI non-redundant protein restricted to *Mus musculus* using Proteome Discoverer version 1.4 (Thermo Fisher Scientific) with the Mascot search engine version 2.5 (Matrix Science). A decoy database comprised of either randomized or reversed sequences in the target database was used for false discovery rate (FDR) estimation, and Percolator algorithm was used to evaluate false positives. Search results were filtered against 1% global FDR for high confidence level. Identified proteins were presented after contaminants detected in the control IgG IP were subtracted from those in HORMAD1 IP.

### Histological analysis

Testes, caudal epididymis and ovaries were fixed in Bouin’s solution, and embedded in paraffin. Sections were prepared on CREST-coated slides (Matsunami) at 6 μm thickness. The slides were dehydrated and stained with hematoxylin and eosin.

For Immunofluorescence staining, testes were embedded in Tissue-Tek O.C.T. compound (Sakura Finetek) and frozen. Cryosections were prepared on the CREST-coated slides (Matsunami) at 8 μm thickness, and then air-dried. The serial sections of frozen testes were fixed in 4% paraformaldehyde in PBS for 5 min at room temperature and washed briefly in PBS. After washing, the serial sections were permeabilized in 0.1% TritonX100 in PBS for 5 min. The sections were blocked in 3% BSA/PBS, and incubated at room temperature with the primary antibodies in a blocking solution. After three washes in PBS, the sections were incubated for 1 h at room temperature with Alexa-dye-conjugated secondary antibodies (1:1500; Invitrogen) in a blocking solution. DNA was counterstained with Vectashield mounting medium containing DAPI (Vector Laboratory).

### Antibody and reagents

Antibodies used in this study were listed in Table 1. Polyclonal antibodies against mouse SYCP2-C (aa 1271–1500) were generated by immunizing rabbits and mice. Polyclonal antibodies against mouse IHO1 (aa 1–574), mouse HORMAD2 (aa 1–306) and mouse HORMAD1 (aa 4–392) were generated by immunizing mice. All His-tagged recombinant proteins were produced by inserting cDNA fragments in-frame with pET28c+ (Novagen) for SYCP2-C, IHO1, HORMAD2, or modified pGEX-4T-2 (Sigma-Aldrich) for HORMAD1 in *E*. *coli* strain BL21-CodonPlus(DE3)-RIPL (Agilent), solubilized in a denaturing buffer (6 M HCl-Guanidine, 20 mM Tris-HCl [pH 7.5]) and purified by Ni-NTA (30210, QIAGEN) under denaturing conditions. The antibodies were affinity-purified from the immunized serum with immobilized antigen peptides on CNBr-activated Sepharose (GE healthcare).

### Immunostaining

Chromatin spreads were prepared as described [36] with modifications. Seminiferous tubules were collected from mice at 8–12 dpp in 300 μL DMEM, and minced using a flathead forceps. Followed by adding additional 700 μL of DMEM, testicular cells were broken apart by sequential steps of pipetting and filtering through a cell strainer (70 μm pore size, BD Falcon). Cells were treated with EdU (10 μM) (C10340, Thermo Fisher Scientific) at 34 °C for 30 min. Cell suspension was then diluted with 24 mL PBS, and centrifuged at 600 × g for 3 min. Cell pellet was resuspended in new 1 mL PBS, and centrifuged again at 600 × g for 5 min. Collected cells were resuspended in 500 μL of 1^st^ hypotonic buffer (1: 1 = PBS: hypotonic buffer [30 mM Tris, 50 mM sucrose, 17 mM citric acid, 5 mM EDTA, 2.5 mM DTT, 0.5 mM PMSF]), and left for 8 min at RT. Treated cells were centrifuged at 600 × g for 5 min, and cell pellet was resuspended in 2^nd^ hypotonic buffer (1: 2 = PBS: 100mM Sucrose) at 2 × 10^4^ cells/μL concentration. A 3 μL aliquot of cell suspension was applied to a slide grass spot containing 20 μL of 1% paraformaldehyde (PFA) in H_2_O. Cells were fixed for 1 h in a humid chamber at RT, and air-dried for 1 hr. Cell preparation was washed in 0.4% DRIWELL (Fujifilm, Japan) in H_2_O over night at 4 °C, and dried for 30 min. The prepared slides were immediately used for immunostaining or stored at -80 °C for further use.

Chromatin fiber preparation was made as described previously [68] with some modifications. Briefly, cell suspension was prepared as described above using mice at 12–25 dpp. For hypotonic treatment, cells were resuspended in 500–1000 μL of 0.8% sodium citrate for 30 min at RT. 250 μL cell suspension was used for centrifugal spreading to the slide using a Cytospin 4 Cytocentrifuge (Thermo Fisher Scientific) at 2000 rpm for 4 min. The slides were immediately immersed in fiber lysis buffer (0.2 M Urea) for 20 min, then fixed in 4% formalin in PBS for 10 min. Followed by permeabilization treatment with 0.1% Triton X-100 in PBS for 10 min, the slides were washed with PBS and immediately used for immunostaining or stored at -80 °C for further use.

Immunofluorescence staining was performed as described previously [16,21]. Briefly, the cell preparation was incubated with 5% BSA in PBS for 30 min at RT, and incubated with the primary antibodies (Table1). Followed by EdU labeling with Alexa 647 according to manufacture’s instruction (C10340, Thermo Fisher Scientific), DNA was counterstained with DAPI (VECTASHIELD H-1200, VECTOR Laboratories, CA, USA). Images were captured with a DeltaVision Elite microscope and images for spread samples were deconvoluted and stacked using a DeltaVision SoftWorx software (Applied Precision).

For super-resolution imaging using a STORM (Stochastic Optical Reconstruction Microscopy) system [69], the cell preparation was incubated with the primary antibodies as above, and the secondary antibodies conjugated with CF 568 (Biotium, 1:200 dilution) or Alexa Fluor 647 (invitrogen, 1:200 dilution) (Table 1). For photoswitching activity of fluorophores, cell preparation was then mounted with a freshly made STORM buffer: a mixture of 7 μL of GLOX solution (glucose oxidase 70 μg/μL [G2133-250KU, Sigma-Aldrich], catalase 3.4 μg/μL [035–12903, Fujifilm, Japan] in 10 mM Tris, pH 8.0 [AM9856, Thermo Fisher Scientific] containing 50 mM NaCl), 70 μL of 1 M cysteamine solution (30070-50G, Sigma-Aldrich) containing 0.74% HCl and 620 μL of 50 mM Tris, pH 8.0 (AM9856, Thermo Fisher Scientific) containing 10 mM NaCl, 10% Glucose. Images were captured using a Nikon Eclipse Ti-E inverted microscope (Nikon Instruments Inc., Japan) equipped with a N-STORM super-resolution microscope system (Nikon Instruments Inc.) and a sCMOS camera ORCA-Flash 4.0 (Hamamatsu Photonics, Japan). Samples were excited with a 561 nm laser for CF 568 and a 640 nm laser for Alexa 640. The fluorescence emission of CF 568 and Alexa Fluor 647 were collected onto an CFI SR HP Apochromat TIRF 100XC Oil lens (Nikon Instruments Inc.). 10,000–15,000 images were recorded and data was acquired using a NIS-Elements imaging software (Nikon Instruments Inc.).

### Proximity ligation assay (PLA)

PLA was performed using Duolink PLA system (Sigma-Aldrich) according to the manufacture’s instruction with some modifications. Briefly, spread chromatin preparations were incubated with the supplied blocking solution for 30 min at 37 °C, and incubated with polyclonal rabbit (anti-HORMAD1) and polyclonal mouse antibodies against RAD21L or REC8 (Table 1) that were diluted in Antibody Diluent buffer over night at RT. The slides were incubated with PLUS and MINUS probes (DUO92001, DUO92005, Sigma-Aldrich) for 100 min at 37 °C, and these probes were then ligated with the Ligation-Ligase (DUO92014, Sigma-Aldrich) for 60 min at 37 °C. The ligated circular DNA was amplified with the Amplification-Polymerase solution that contains complementary detection oligonucleotide probes labeled with a fluorophore (DUO92014, Sigma-Aldrich) for 160 min at 37 °C. *In situ* wash buffer (DUO82049, Sigma-Aldrich) was used between each step according to the manufacture’s instruction. The slides were then incubated with polyclonal rat antibody against SYCP3 (Table 1) over night at RT and Alexa-568-labeled secondary antibody (1:1000 dilution, invitrogen) in the blocking solution for 1 h at RT, followed by EdU labeling with Alexa 647 (C10340, Thermo Fisher Scientific) as above. The preparation slides were mounted with a Duolink *in situ* mounting medium with DAPI (DUO8240, Sigma-Aldrich).

### Co-localization analysis

Colocalization of two different proteins in immunostaining was analyzed using the ComDet v.0.4.1 plugin (https://github.com/ekatrukha/ComDet) of Fiji software version 2.0.0 [70]. Dotty signals of each protein were detected separately in each channel at approximately 3 pixels in size for HORMAD1/RAD21L/REC8 immunostained dotty signals in preleptotene cells, or 5 pixels in size for IHO1 dotty signals in leptotene (-like) cells. SMC3 threads in leptotene cells were detected as segmented particles at approximately 4 pixels in size. Co-localization was defined based on the maximum distance between the centers of two dots at the threshold of 1, 3 or 5 pixels depending on the assay. Colocalization of two different proteins was quantified in percentage.

### Statistical analyses

Multiple biological replicates from two or more animals for each strain were analyzed for the phenotypes. The significance of HORMAD1/RAD21L/REC8 dot counting and pairing assay was assessed by ordinary one-way Analysis of variance (ANOVA) with Tukey’s correction for multiple test using GraphPad Prism7 software.

## Supporting information

S1 Fig Generation of *Sycp2-*KO mouse. **(A)** WB analysis of testis extracts prepared from WT and *Sycp2*-KO mice (P18). The testis extracts were run on 4–12% NuPAGE Bis-Tris in MOPS-SDS buffer. Immunoblots of axis-associated proteins are shown. **(B)** Hematoxylin and eosin staining of the sections from WT and *Sycp2-*KO testes (eight-week-old). Scale bar: 100 μm. **(C)** Hematoxylin and eosin staining of the sections from WT and *Sycp2*-KO mice epididymis (eight-week-old). Scale bar: 100 μm.
(TIF)

S2 Fig Chromosome axis localization of HORMAD1 and RAD21L in mutant mice. Spread chromatin of spermatocytes from WT, *Rad21L*-KO, *Rec8*-KO, *Spo11*-KO and *Hormad1*-KO mice were immunolabelled for HORMAD1 (magenta) and RAD21L (green). Scale bar: 10 μm.
(TIF)

S3 Fig Chromosome axis localization of HORMAD1 and REC8 in mutant mice. Spread chromatin of spermatocytes from WT, *Rad21L*-KO, *Rec8*-KO, *Spo11*-KO and *Hormad1*-KO mice were immunolabelled for HORMAD1 (magenta) and REC8 (green). Scale bar: 10 μm.
(TIF)

S1 Data Raw data of LC-MSMS analyses. The LC-MSMS raw data of control IgG IP, HORMAD1 IP, REC8 IP and RAD21L IP are shown. Whole proteins are listed, that were identified in control IgG IP, HORMAD1 IP, REC8 IP or RDA21L IP by LC-MSMS analyses. Proteins after subtraction of those identified in control IgG IP and IgG fragment itself are listed in a separate tab.
(XLSX)
