# Supplementary figures and images for: Meiotic cohesins mediate initial loading of HORMAD1 to the chromosomes and coordinate SC formation during meiotic prophase
Source: PLoS Genet. 2020 Sep 15;16(9):e1009048. doi: 10.1371/journal.pgen.1009048 (PMC7518614; doi:10.1371/journal.pgen.1009048)

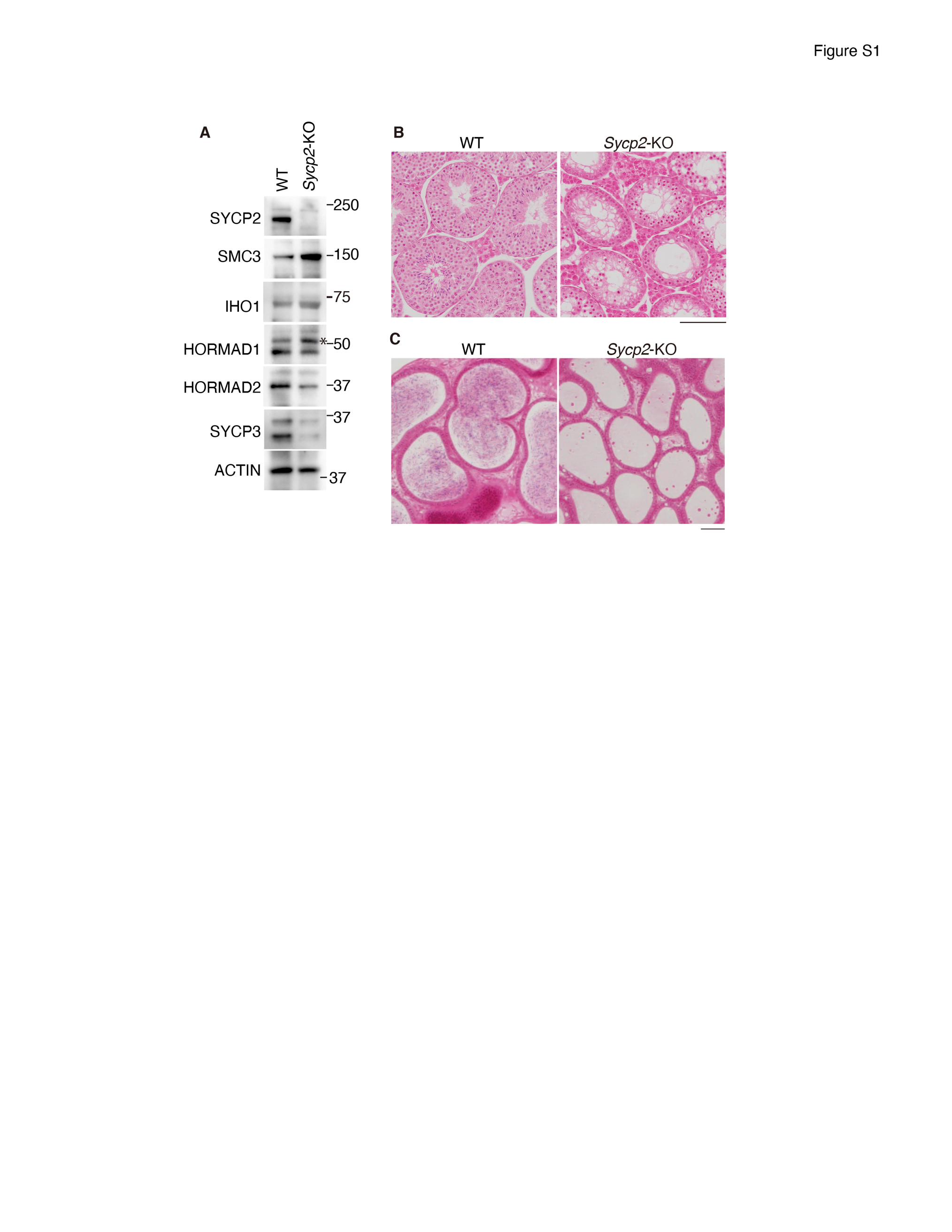

Supplement: S1 Fig — (A) WB analysis of testis extracts prepared from WT and Sycp2-KO mice (P18). The testis extracts were run on 4–12% NuPAGE Bis-Tris in MOPS-SDS buffer. Immunoblots of axis-associated proteins are shown. (B) Hematoxylin and eosin staining of the sections from WT and Sycp2-KO testes (eight-week-old). Scale bar: 100 μm. (C) Hematoxylin and eosin staining of the sections from WT and Sycp2-KO mice epididymis (eight-week-old). Scale bar: 100 μm. (TIF) [file pgen.1009048.s001.tif]

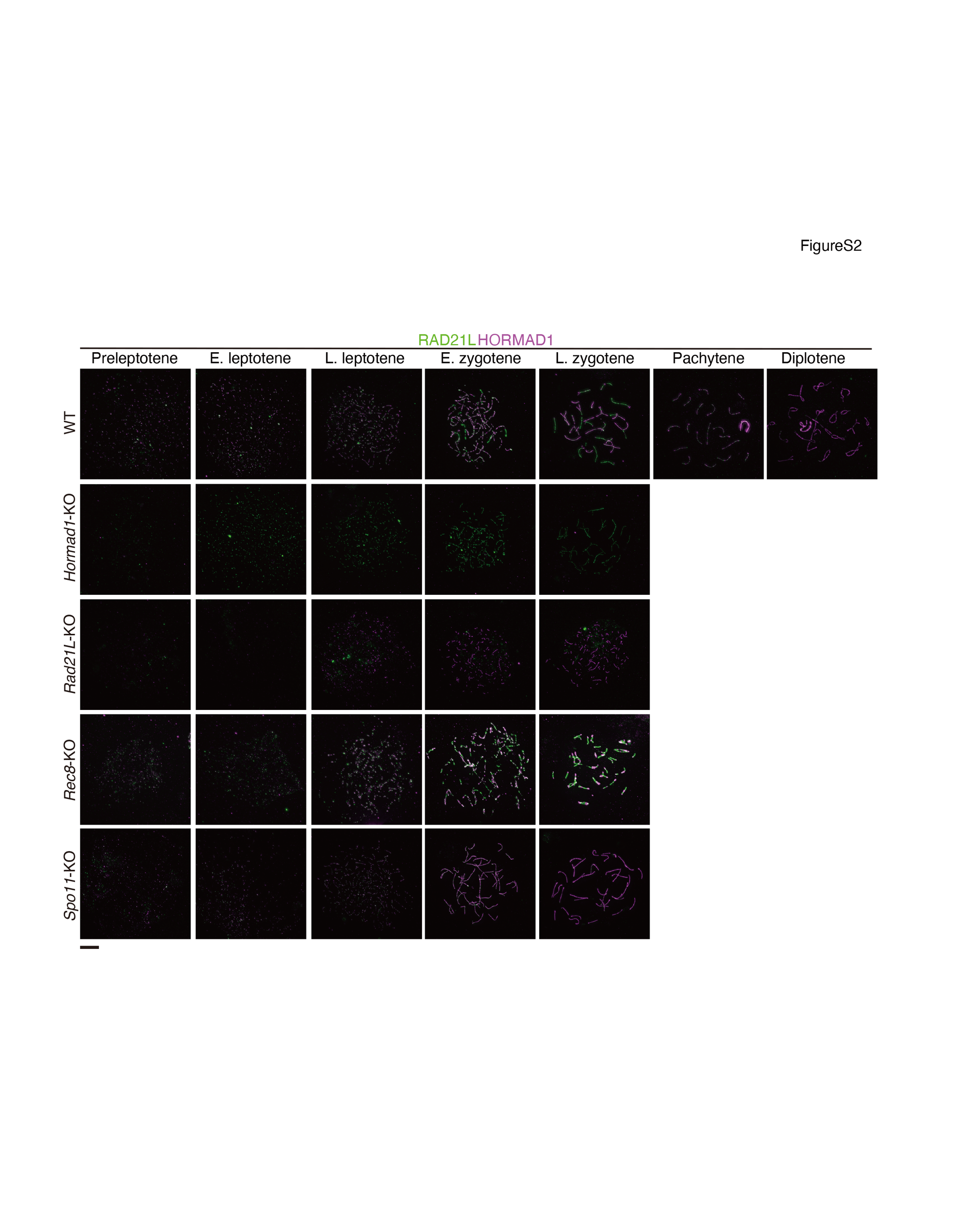

Supplement: S2 Fig — Spread chromatin of spermatocytes from WT, Rad21L-KO, Rec8-KO, Spo11-KO and Hormad1-KO mice were immunolabelled for HORMAD1 (magenta) and RAD21L (green). Scale bar: 10 μm. (TIF) [file pgen.1009048.s002.tif]

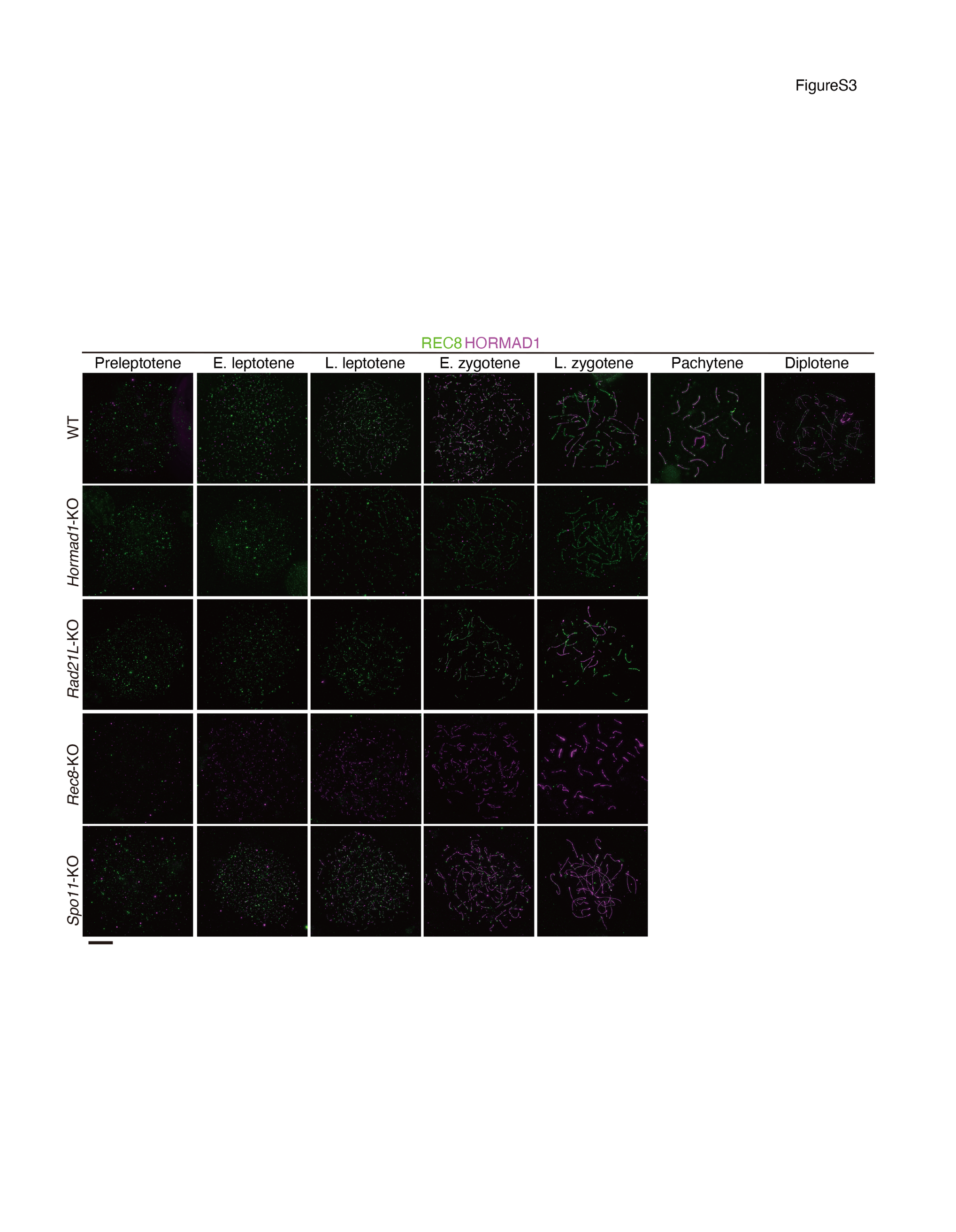

Supplement: S3 Fig — Spread chromatin of spermatocytes from WT, Rad21L-KO, Rec8-KO, Spo11-KO and Hormad1-KO mice were immunolabelled for HORMAD1 (magenta) and REC8 (green). Scale bar: 10 μm. (TIF) [file pgen.1009048.s003.tif]
